# Supplementary material for: Pulse-controlled qubit in semiconductor double quantum dots
Source: Sci Rep. 2023 Dec 4;13:21369. doi: 10.1038/s41598-023-47405-0 (PMC10695949; doi:10.1038/s41598-023-47405-0)
Supplement: Supplementary file 1 — Supplementary Information. [file 41598_2023_47405_MOESM1_ESM.pdf]

Supplementary Information

I. READOUT

For completeness, we discuss a potential procedure for the readout process. The below results justify using the superpositions of the first two energy states as logical qubits - as opposed to a more general operation that strongly localizes the wave function to one side of a double quantum dot. We show here that even though a qubit in the left dot has nonzero probability of being found to the right of center, the qubit can still be read out reliably. In experimental setups, it is the probability of finding the electron in one of the dots which is measured rather than the qubit superposition weighting coefficients. We can express both qubits defined in the main text in terms of their right and left dots parts:

$$\psi_0(x) = \langle x|0\rangle = f_{0L}(x) + f_{0R}(x) \tag{1}$$
$$\psi_1(x) = \langle x|1\rangle = f_{1L}(x) + f_{1R}(x) \tag{2}$$

Because the qubits $|0\rangle$ and $|1\rangle$ are orthogonal, we have:

$$\begin{aligned} 0 = \int \psi_0^*(x)\psi_1(x)dx = \int f_{0L}^*(x)f_{1L}(x)dx + \\ \int f_{0L}^*(x)f_{1R}(x)dx + \int f_{0R}^*(x)f_{1L}(x)dx + \\ \int f_{0R}^*(x)f_{1R}(x)dx = \int f_{0L}^*(x)f_{1L}(x)dx + \\ \int f_{0R}^*(x)f_{1R}(x)dx. \end{aligned} \tag{3}$$

The qubits are mirror images of each other, such that $\langle x|0\rangle$ has the same spatial distribution in the left (right) dot as $\langle x|1\rangle$ has in the right (left) one. We also know that there is some non-zero overlap, unless the double quantum dot (DQD) barrier is completely separating the dots. Therefore Eq. 3 implies that :

$$\int f_{0R}^*(x)f_{1R}(x)dx = -\int f_{0L}^*(x)f_{1L}(x)dx = \eta. \tag{4}$$

Any arbitrary state can be written as a linear combination of the two qubits right and left dot components

$$\begin{aligned} \psi(x) = \alpha\psi_0(x) + \beta\psi_1(x) = \\ \alpha\Big(f_{0L}(x) + f_{0R}(x)\Big) + \beta\Big(f_{1L}(x) + f_{1R}(x)\Big), \end{aligned} \tag{5}$$

The probability P_R of finding the particle in the right dot is then:

$$\begin{aligned} P_R = \int_0^\infty \psi^*(x)\psi(x)dx = \int_0^\infty \Big(\alpha^* f_{0R}^*(x) + \\ \beta^* f_{1R}^*(x)\Big)\Big(\alpha f_{0R}(x) + \beta f_{1R}(x)\Big)dx. \end{aligned} \tag{6}$$

Using Eq. 4, this reduces to:

$$\begin{aligned} P_R = |\alpha|^2 \int_0^\infty f_{0R}^*(x)f_{0R}(x)dx + \\ |\beta|^2 \int_0^\infty f_{1R}^*(x)f_{1R}(x)dx + \eta(\alpha^*\beta + \alpha\beta^*) = \\ |\alpha|^2 P_{0R} + |\beta|^2 P_{1R} + 2\eta\mathscr{R}(\alpha^*\beta), \end{aligned} \tag{7}$$

where the integrals P_{0R} and P_{1R} can be obtained initialising the qubit in the $\psi_0(x)$ or $\psi_1(x)$ state, respectively, and measuring the probability of finding it in the right dot. Combining Eq. 7 with the normalisation condition for $\psi(x)$, we obtain an equation relating $|\beta|$ to the probability P_R of finding the particle in the right dot, up to an error term proportional to η, which quantifies the uncertainty of determining whether the qubit is in the left or right side of the DQD:

$$|\beta|^2 = \frac{P_R - P_{0R}}{P_{1R} - P_{0R}} + \delta. \tag{8}$$

A similar expression exists for $|\alpha|^2$, with P_L being the probability of finding the particle in the left dot :

$$|\alpha|^2 = \frac{P_L - P_{0R}}{P_{1R} - P_{0R}} - \delta, \tag{9}$$

where $\delta = 2\eta\frac{\mathscr{R}(\alpha^*\beta)}{P_{0R} - P_{1R}}$ is the effective error. Since $P_{1R} \approx 1$, $P_{0R} \approx 0$, we can estimate the maximum readout error, which would occur for a maximally entangled state:

$$\mid \delta \mid \lessapprox \eta. \tag{10}$$

For the parameters used in this paper, $\mid \delta \mid \leq 8 \cdot 10^{-4}$. This magnitude of readout error is not very significant compared to other sources of errors in a quantum computation [1, 2], such as two-qubit gates, relaxation, or dephasing, especially since it's only applied once as the final step. Additionally, it was shown [3] that in a similar situation, adiabatically increasing the inter-dot barrier of the DQD preserves coherence, while greatly reducing this type of "overlap" error -this technique should be used when possible if the readout error is noticable. Alternatively, as this error is a result of lack of knowledge of $\mathscr{R}(\alpha^*\beta)$, a full state tomography could be performed to eliminate it completely (assuming that errors of operations associated with the tomography do not outweigh the readout error). Therefore, we conclude that measurement of the charge distribution is a viable way of reading out the qubit in our scheme.

II. TWO-SITE LOCALISED STATE MODEL AND DQD POTENTIAL

Within the two-state model, one has to solve the time dependent Schrödinger equation (TDSE) with the effective Hamiltonian $\hat{H}_{\text{eff}}$ defined as

$$\hat{H}_{\text{eff}}(t) = -\frac{1}{2}\,\epsilon(t)\,\sigma_x + \frac{1}{2}\,\Delta\,\sigma_z + \frac{1}{2}(E_{\text{B}} + E_{\text{AB}}). \quad (11)$$

Here E_{B} and E_{AB} are the energies of the bonding and antibonding states of the DQD system, i.e. the two lowest energy states, at $\epsilon = 0$ whereas Δ is the 'hybridisation energy' between the two localised states. At zero detuning, the bonding state $\psi^{\text{B}}(x)$ is symmetric, while the antibonding state $\psi^{\text{AB}}(x)$ is antisymmetric. Therefore, their equal superpositions produce maximally localised left/right states:

$$\psi^{L}(x) = \frac{1}{\sqrt{2}}(\psi^{\text{B}}(x) + \psi^{\text{AB}}(x)), \quad (12)$$

$$\psi^{R}(x) = \frac{1}{\sqrt{2}}(\psi^{\text{B}}(x) - \psi^{\text{AB}}(x)). \quad (13)$$

The linear detuning breaks the left/right symmetry, however as it is expressed by the Pauli σ_x matrix in the Hamiltonian, it doesn't make the system leave the $\psi^{\text{B}}(x)/\psi^{\text{AB}}(x)$ two-state basis (if done adiabatically), resulting simply in a coordinate rotation of the Bloch sphere. Therefore, we can still think in terms of the left-/right localised wave functions even at non-zero detuning, and varying ϵ is a viable way of performing single qubit rotations. The two-site localised state model describes a DQD well. The effective potential in an experimental DQD system can be found using density functional theory [4, 5] and will be a complex function of all three spatial coordinates x, y, z. By careful design, the dynamics in two of the directions y and z can be confined to the lowest energy subbands so that only the potential in the x direction, $V_{\text{DQD}}(x,t)$ needs be considered. For example in a GaAs/AlGaAs heterostructure, the z direction is the growth direction and modulation doping can be used to create a triangular quantum well in that direction with subband energies two orders of magnitude larger than either ϵ or Δ. In the y direction, parabolic confinement with energies an order of magnitude larger than ϵ or Δ can be produced either by etching [6], fabricating a thin gate wrapping the conducting channel [7, 8] or using split-gates [9]. In order to create a DQD potential in the x direction, gates [10–13] or etching [6, 14] can also be used.

The aim is to create a potential $V_{\text{DQD}}(x,t)$ that has two minima separated by a tunnel barrier. A convenient potential that has this property and is defined by three parameters A, B and σ is given by

$$V_{\text{DQD}}(x) = Ax^2 + B\exp\left(\frac{-x^2}{2\sigma}\right) \quad (14)$$

This form for V_{DQD} allows us to control both the depth of the dots and the barrier between them directly, by varying the harmonic confinement A, barrier height B, and barrier width σ. This potential will obey the two-site localised state model. For a specific set of parameters, this static potential will define a value for Δ which is the energy difference between the bonding ground state E_{B} and the antibonding first excited state E_{AB}. Detuning is introduced by adding a linear Stark shift of the form

$$V_{\text{linear}}(x) = V_{\text{bias}}\frac{x}{2w}. \quad (15)$$

Here, w is half the width of the DQD. By comparing the dependences of E_{B} and E_{AB} on V_{bias} with the expected dependences from two-site Hamiltonian we can define the detuning parameter for V_{DQD} through a linear relation $\epsilon = e\lambda V_{\text{bias}}$ with λ being constant. We find this linear relationship holds with an accuracy of one part in 10^6 across the range of required values of ϵ for single-qubit operations. The total potential is $V_{\text{tot}}(x) = V_{\text{DQD}}(x) + V_{\text{linear}}(x)$ and Fig. 1a shows this potential at three different detunings.

The DQD dynamics under time-dependent detuning will be given by the TDSE

$$\hat{H}(x,t)\psi(x,t) = i\hbar\frac{\partial}{\partial t}\psi(x,t) \quad (16)$$

with

$$\hat{H}(x,t) = -\frac{\hbar^2}{2m^*}\frac{\partial^2}{\partial x^2} + V_{\text{DQD}}(x) + V_{\text{bias}}(x,t). \quad (17)$$

Time dependence is included in Eq. 16 by varying the potential slope with time: $V_{\text{bias}}(t)$. An example plot of the energies of the two lowest instantaneous solutions (the bonding and antibonding states) as function of V_{bias} is shown in Fig. S1 (b).

Analytic solutions to the TDSE in Eq. 17 can only be found in special cases. In this paper we solve Eq. 16 numerically using a GPU-accelerated version of the staggered-leapfrog method [15, 16] (see App. IV).

Throughout the paper we avoid using specific numerical values to keep our results general. However, here we give the actual values used for reproducibility. We've use a total DQD length of 460 nm, with parameter values: $w = 230$ nm, $A = 1.276$ meV nm^{-1}, $B = 4.08$ meV so that $\mathit{\Delta} = 11.7\mu$eV, and the linear coefficient $\lambda = 0.421$. We have also tested various non-symmetric potentials with the two dots having different sizes, but in all the cases the general conclusions were the same as for the symmetric potential of Eq. 14.

III. ROTATION SCHEME DERIVATION

We find a fast and simple general rotation scheme based on creating two perpendicular axes $\vec{x}'$ and $\vec{z}'$, by

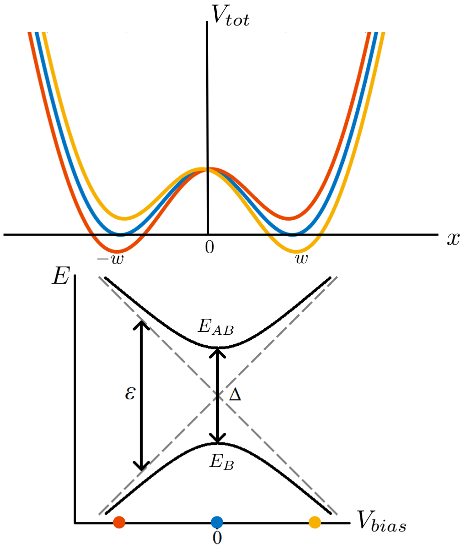

Supplementary Fig. S 1. (a) The DQD potential V_{tot} at zero (blue), lowest (red) and highest (orange) detuning values. (b) Energies E of the bonding (E_{B}) and anti-bonding (E_{AB}) eigenstates. The coloured dots mark potential shapes from part (a).

setting the detuning $\epsilon = \pm\Delta$. Then we observe that one should be able to perform a rotation around an axis at $\frac{\pi}{4}$ w.r.t the two axes above, which would be $\vec{x}$ and $\vec{z}$. This is achieved by rotating by some angle Θ_1 around the first axis, then by Θ_2 around the second one, and finally by Θ_1 around the first one again.

We will find the relationship between Θ_1, Θ_2, and the net angle rotated around $\vec{x}$ or $\vec{z}$ named α, by analytically comparing the rotation matrix elements with the straightforward $R_{\vec{x}}$ and $R_{\vec{z}}$ rotations.

Looking at $R_{\vec{x}}$ first:

$$R_{\vec{x}}(\alpha) = \begin{pmatrix} \cos\frac{\alpha}{2} & -i\sin\frac{\alpha}{2} \\ -i\sin\frac{\alpha}{2} & \cos\frac{\alpha}{2} \end{pmatrix}. \tag{18}$$

In our scheme,

$$R_{\vec{x'}}(\alpha) = R_{\vec{y}}\left(\frac{-\pi}{4}\right) R_{\vec{x}}(\alpha) R_{\vec{y}}\left(\frac{\pi}{4}\right), \tag{19}$$

$$R_{\vec{z'}}(\alpha) = R_{\vec{y}}\left(\frac{-\pi}{4}\right) R_{\vec{z}}(\alpha) R_{\vec{y}}\left(\frac{\pi}{4}\right), \tag{20}$$

and we need the following to always hold:

$$R_{\vec{x}}(\alpha) = R_{\vec{x'}}(\Theta_1) R_{\vec{z'}}(\Theta_2) R_{\vec{x'}}(\Theta_1). \tag{21}$$

Comparing the (1,1) matrix elements:

$$\begin{aligned} \cos\alpha = \cos\frac{\Theta_2}{2}(2\cos^2\frac{\Theta_1}{2} - 1) \\ -j\sqrt{2}(\frac{1}{2}\sin\frac{\Theta_2}{2} + \cos\frac{\Theta_1}{2}\cos\frac{\Theta_2}{2}\sin\frac{\Theta_1}{2}). \end{aligned} \tag{22}$$

Since the imaginary part on the LHS is zero, we have:

$$(\frac{1}{2}\sin\frac{\Theta_2}{2} + \cos\frac{\Theta_1}{2}\cos\frac{\Theta_2}{2}\sin\frac{\Theta_1}{2}) = 0. \tag{23}$$

Solving the above allows us to find Θ_2 in terms of Θ_1:

$$\Theta_2 = 2\arctan(\sin\Theta_1). \tag{24}$$

Now coming back to the real part of Eq. 22 and substituting for Θ_2, we have:

$$\cos(\arctan(\sin\Theta_1))\cos\Theta_1 = \cos\frac{\alpha}{2}, \tag{25}$$

which gives

$$\Theta_1 = \arccos\left(\frac{\sqrt{2}\cos\frac{\alpha}{2}}{\sqrt{\cos\frac{\alpha}{2}^2 + 1}}\right). \tag{26}$$

The above satisfies Eq. 21 for all matrix elements, and is therefore equivalent. It allows us to find a three pulse train that performs the $R_{\vec{x}}(\alpha)$ rotation by an arbitrary angle α. We repeat the above procedure for $R_{\vec{z}}$ to find the following:

$$\Theta_2 = -2\arctan(\sin\Theta_1) + 2\pi, \tag{27}$$

$$\Theta_1 = \arccos\left(\frac{\sqrt{2}\cos\frac{\alpha}{2}}{\sqrt{\cos\frac{\alpha}{2}^2 + 1}}\right). \tag{28}$$

Therefore, we can perform arbitrary rotations around $\vec{z}$ and $\vec{x}$ this way. However, this scheme is unable to perform the $R_{\vec{y}}$ rotation, which is achieved differently, as described in the main text.

IV. ITERATION METHOD

The system is modelled using an explicit iterative scheme for the one-dimensional time-dependent Schrödinger equation with an arbitrary potential *V(x,t)*:

$$i\hbar\frac{\partial\psi(x,t)}{\partial t} = H\psi = \left[\frac{-\hbar^2}{2m}\frac{\partial^2}{\partial x^2} + V(x,t)\right]\psi(x,t) \tag{29}$$

where m is the effective mass. The scheme, which is based on the finite difference method, was described in details by Maestri *et al.* for two particles in one dimension [17] and we adapt it to a single particle. The wave function is evaluated on a spatially discretized grid and at successive, equally separated intervals of time Δt:

$$\psi(x,t) = \psi(m\Delta x, k\Delta t) \equiv \psi_m^k, \tag{30}$$

with m, k integer. The spatial part of the method is derived using Taylor expansion of the wave function:

$$\frac{\partial^2\psi}{\partial x^2} \simeq \frac{\psi(x+\Delta x) - 2\psi(x) + \psi(x-\Delta x)}{\Delta x^2}. \quad (31)$$

Therefore, using Eqs. (30) and (31), the right hand side of Eq. (29) transforms into

$$H\psi = \left[\frac{-\hbar^2}{2m}\left(\frac{\psi_{m+1} - 2\psi_m + \psi_{m-1}}{\Delta x^2}\right) + V_m\right]\psi_m. \quad (32)$$

The derivative on the left hand side of Eq. (29) is calculated by writing the exact solution of TDSE and then taking the difference between the $(k+1)^{th}$ and $(k-1)^{th}$ time steps, as suggested by Askar and Cakmak [15]:

$$\psi_m^{k+1} = e^{-i\Delta t H/\hbar}\psi_m^k \simeq \left(1 - \frac{i\Delta t H}{\hbar}\right)\psi_m^k, \quad (33)$$

$$\psi_m^{k+1} - \psi_m^{k-1} = (e^{-i\Delta t H/\hbar} - e^{i\Delta t H/\hbar})\psi_m^k \simeq -\frac{2i\Delta t H}{\hbar}\psi_m^k. \quad (34)$$

To improve the accuracy, we follow Visscher's staggered-time method [18] and write the wave vector in terms of its real and imaginary parts: $\psi_m^k = u_m^k + iv_m^k$. After inserting the Hamiltonian from Eq. (32) into Eq. (34) and rearranging the terms, we obtain a pair of simultaneous equations, which are iterated over time:

$$u_m^{k+1} = u_m^{k-1} + \left(2a_x + b\,V_m^k\right)v_m^k - \quad (35)$$

$$a_x(v_{m+1}^k + v_{m-1}^k), \quad (36)$$

$$v_m^{k+1} = v_m^{k-1} - \left(2a_x + b\,V_m^k\right)u_m^k - \quad (37)$$

$$a_x(u_{m+1}^k + u_{m-1}^k), \quad (38)$$

where $a_x = \frac{\hbar\Delta t}{m\Delta x^2}$ and $b = \frac{2\Delta t}{\hbar}$. Also, the real and imaginary parts are calculated at slightly shifted times: $u^k \equiv u(t)$, $v^k \equiv v(t+\Delta t/2)$.

The method above is stable as long as the following criterion is satisfied:

$$\Delta t \leq \frac{\hbar}{E_{\max}}, \quad (39)$$

with $E_{\max}$ being the largest eigenvalue of the discretised Hamiltonian [19]. Furthermore, small errors due to finite computational accuracy do not accumulate with iterations and the total electron probability $\sum_{\text{all } m}|\psi_m^k|^2$ is preserved over time, showing no significant deviations from unity.

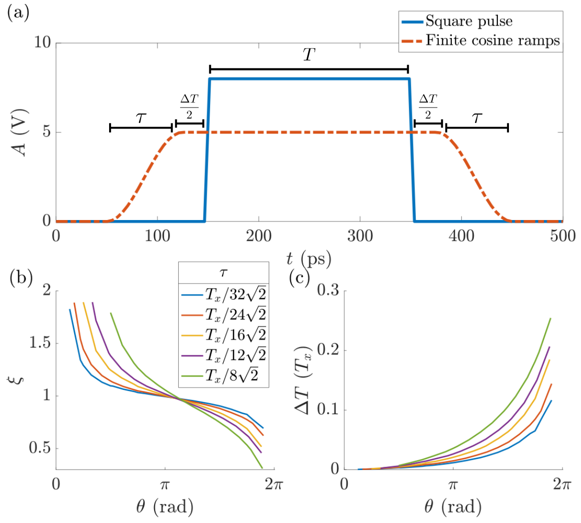

Supplementary Fig. S 2. **(a)** Amplitude profile of the ideal square pulse (solid blue), and the pulse adjusted for rise time (dotted red). The pulse amplitude and duration are adjusted when the rise time τ is finite. Values of time (pulse duration, rise time) and voltage (pulse amplitude) are given for illustration purposes only. **(b)** Multiplicative amplitude adjustment factor ξ given a target rotation angle θ. Each coloured line corresponds to a different τ (see legend). **(c)** Additive pulse duration adjustment ΔT with respect to the original square pulse time (see panel **(a)**). The rise times are not included in the additional pulse duration. Each coloured line corresponds to a different τ (see legend of **b**).

V. FINDING OPTIMAL ADJUSTMENT PARAMETERS ACCOUNTING FOR RISE TIME τ BY GRADIENT ASCENT

To adjust a square pulse for rise time τ, we apply a gradient ascent algorithm in conjunction with numerical simulations. To illustrate the parameter space, we reproduce a figure from the main text as Fig. S2. For a given value of τ and Θ (desired rotation angle), the algorithm searches a two-dimensional space of ΔT and ξ. An adjusted pulse is applied k times to an initial state $\psi = \frac{1}{\sqrt{2}}(|0\rangle + |1\rangle)$. The cost function is the error: 1 - fidelity. The fidelity is calculated as the absolute value squared of the overlap between the resulting state, and the state we expect after a perfect rotation on the Bloch sphere. Chaining the pulse $k = 2-4$ times improves precision of parameters found.

Because the rotation scheme is general by design, the same pulse will achieve the same rotation angle, (around the $(\frac{1}{\sqrt{2}}, 0, \frac{1}{\sqrt{2}})$ axis on the Bloch sphere) irrespective of the initial state. From these primary pulses, an arbitrary rotation can be constructed and optimized for total time, as described in detail in the main text.

The MATLAB code for finding the optimal adjustment parameters accounting for rise time τ for single qubit control is available on request from the corresponding author. The time-dependent evolution is relegated to the

GPU-accelerated staggered-leapfrog code described and referenced in the main work.

[1] S. Li, A. D. Castellano, S. Wang, Y. Wu, M. Gong, Z. Yan, H. Rong, H. Deng, C. Zha, C. Guo, L. Sun, C. Peng, X. Zhu, and J.-W. Pan, npj Quantum Information **5**, 84 (2019).

[2] W. Huang, C. H. Yang, K. W. Chan, T. Tanttu, B. Hensen, R. C. C. Leon, M. A. Fogarty, J. C. C. Hwang, F. E. Hudson, K. M. Itoh, A. Morello, A. Laucht, and A. S. Dzurak, Nature **569**, 532 (2019).

[3] H. V. Lepage, A. A. Lasek, D. R. M. Arvidsson-Shukur, and C. H. W. Barnes, Phys. Rev. A **101**, 022329 (2020).

[4] E. T. Owen and C. H. W. Barnes, Phys. Rev. Appl. **6**, 054007 (2016).

[5] M. Stopa, Phys. Rev. B **54**, 13767 (1996).

[6] T. Ferrus, A. Rossi, M. Tanner, G. Podd, P. Chapman, and D. A. Williams, New J. Phys. **13**, 103012 (2011).

[7] D. Hisamoto, T. Kaga, Y. Kawamoto, and E. Takeda, IEEE Electron Device Letters **11**, 36 (1990).

[8] B. Voisin, V.-H. Nguyen, J. Renard, X. Jehl, S. Barraud, F. Triozon, M. Vinet, I. Duchemin, Y.-M. Niquet, S. de Franceschi, and M. Sanquer, Nano Lett. **14**, 2094 (2014).

[9] B. J. van Wees, H. van Houten, C. W. J. Beenakker, J. G. Williamson, L. P. Kouwenhoven, D. van der Marel, and C. T. Foxon, Phys. Rev. Lett. **60**, 848 (1988).

[10] W. G. van der Wiel, S. D. Franceschi, J. M. Elzerman, T. Fujisawa, S. Tarucha, and L. P. Kouwenhoven, Rev. Mod. Phys. **75**, 1 (2002).

[11] S. Gardelis, C. G. Smith, J. Cooper, D. A. Ritchie, E. H. Linfield, Y. Jin, and M. Pepper, Phys .Rev. B **67**, 073302 (2003).

[12] W. H. Lim, H. Huebl, L. H. W. van Beveren, S. Rubanov, P. G. Spizzirri, S. J. Angus, R. G. Clark, and A. S. Dzurak, Appl. Phys. Lett. **94**, 173502 (2009).

[13] N. Mason, M. J. Biercuk, and C. M. Marcus, Science **303**, 655 (2004).

[14] D. Wei, H.-O. Li, G. Cao, G. Luo, Z.-X. Zheng, T. Tu, M. Xiao, G.-C. Guo, H.-W. Jiang, and G.-P. Guo, Sci. Rep. **3**, 2323 (2013).

[15] A. Askar and A. S. Cakmak, The Journal of Chemical Physics **68**, 2794 (1978).

[16] E. Owen, M. Dean, and C. Barnes, Physical Review A **85**, 022319 (2012).

[17] J. J. V. Maestri, R. H. Landau, and M. J. Paez, American Journal of Physics **68**, 1113 (2000).

[18] P. B. Visscher, Comput. Phys. **5**, 596 (1991).

[19] C. Leforestier, R. H. Bisseling, C. Cerjan, M. D. Feit, R. Friesner, A. Guldberg, A. Hammerich, G. Jolicard, W. Karrlein, H.-D. Meyer, N. Lipkin, O. Roncero, and R. Kosloff, J. Comp. Phys. **94**, 59 (1991).
